# Adrenergic Signaling-Induced Ultrastructural Strengthening of Intercalated Discs via Plakoglobin Is Crucial for Positive Adhesiotropy in Murine Cardiomyocytes

**DOI:** 10.3389/fphys.2020.00430

**Published:** 2020-05-21

**Authors:** Sunil Yeruva, Ellen Kempf, Desalegn Tadesse Egu, Heinrich Flaswinkel, Daniela Kugelmann, Jens Waschke

**Affiliations:** ^1^Institute of Anatomy and Cell Biology, Faculty of Medicine, Ludwig-Maximilians-Universität Munich, Munich, Germany; ^2^BioSysM, Ludwig-Maximilians-Universität Munich, Munich, Germany

**Keywords:** desmosome, arrhythmogenic cardiomyopathy, intercalated disc, intercellular adhesion, positive adhesiotropy

## Abstract

Intercalated discs (ICDs), which connect adjacent cardiomyocytes, are composed of desmosomes, adherens junctions (AJs) and gap junctions (GJs). Previous data demonstrated that adrenergic signaling enhances cardiac myocyte cohesion, referred to as positive adhesiotropy, via PKA-mediated phosphorylation of plakoglobin (PG). However, it was unclear whether positive adhesiotropy caused ultrastructural modifications of ICDs. Therefore, we further investigated the role of PG in adrenergic signaling-mediated ultrastructural changes in the ICD of cardiomyocytes. Quantitative transmission electron microscopy (TEM) analysis of ICD demonstrated that cAMP elevation caused significant elongation of area composita and thickening of the ICD plaque, paralleled by enhanced cardiomyocyte cohesion, in WT but not PG-deficient cardiomyocytes. STED microscopy analysis supported that cAMP elevation *ex vivo* enhanced overlap of desmoglein-2 (Dsg2) and N-cadherin (N-cad) staining in ICDs of WT but not PG-deficient cardiomyocytes. For dynamic analyses, we utilized HL-1 cardiomyocytes, in which cAMP elevation induced translocation of Dsg2 and PG but not of N-cad to cell junctions. Nevertheless, depletion of N-cad but not of Dsg2 resulted in a decrease in basal cell cohesion whereas positive adhesiotropy was abrogated in monolayers depleted for either Dsg2 or N-cad. In the WT mice, ultrastrutural changes observed after cAMP elevation were paralleled by phosphorylation of PG at serine 665. Our data demonstrate that in murine hearts adrenergic signaling enhanced N-cad and Dsg2 in the ICD paralleled by ultrastrutural strengthening of ICDs and that effects induced by positive adhesiotropy were strictly dependent on Pg.

## Introduction

The intercalated disc (ICD) is composed of desmosomes, adherens junctions (AJs), and gap junctions (GJs), provides electrical conduction and mechanical stability to the cardiomyocytes and ultimately to the heart (Waschke, 2008). Adjacent cardiomyocytes are connected by members of the cadherin family, which span the intercellular cleft and are linked to several plaque proteins and the cytoskeleton. Cardiomyocytes express desmosomal cadherins desmoglein-2 (Dsg2) and desmocollin-2 (Dsc2) and the AJ protein N-cadherin (N-cad) (Franke et al., 2006), which are all targeted to ICDs where they bind extracellularly in Ca^2+^-dependent manner to their respective partners of the adjacent cardiac myocyte (Al-Amoudi and Frangakis, 2008). On the other hand, the cytosolic part of these adhesion molecules is connected to the cytoskeletal network via the plaque proteins plakoglobin (PG), plakophilin (PKP), and desmoplakin (DP) in desmosomes or to β-catenin and other plaque proteins in AJ, respectively. In cardiomyocytes, components of desmosomes are not separated strictly but intermingled, referred to as the area composita (Franke et al., 2006; Pieperhoff and Franke, 2007).

Arrhythmogenic cardiomyopathy (ACM) is a heart disease associated with arrhythmia, which results in sudden cardiac death of young individuals (Corrado et al., 2017). ACM is characterized by a deficiency in mechanical and electrical communication among cardiomyocytes. ACM is known to be caused by mutations in desmosomal proteins such as Dsg2, Pkp, PG, and DP in up to 60% cases (Corrado et al., 2017). However, recently, two studies highlighted that mutations in CDH2 gene (N-cad protein) are also present in ACM patients, showing the importance of AJ proteins in the outcome of ACM (Mayosi et al., 2017; Turkowski et al., 2017; Karmouch et al., 2018). Even though the pathology of this disease is well characterized, less is known about how the interaction of desmosomal and AJ proteins can lead to ventricular arrhythmia.

The sympathetic nervous system acts through β-adrenergic receptors in cardiomyocytes and is known to increase heart rate. Recently, we have provided the first evidence of β-adrenergic signaling enhancing cardiomyocyte cell cohesion, which we referred to as positive adhesiotropy (Schinner et al., 2017). This effect was dependent on PG and its phosphorylation at S665 by PKA (Schinner et al., 2017). In this study, we further assessed ultrastructural changes in ICD of murine cardiomyocytes by adrenergic signaling and provide the first evidence that adrenergic signaling-mediated positive adhesiotropy depends on the ultrastructural strengthening of ICDs via PG.

## Materials and Methods

### Ethical Approval

Handling, breeding, and sacrificing of mice for scientific purposes was performed under approval of regulations of the Regierung von Oberbayern (Gz.: 55.2-1-54-2532-139-2014).

### Mediators

The combination of the adenylyl cyclase activator forskolin and phosphodiesterase-4 inhibitor rolipram (F/R, Sigma–Aldrich, #F3917/#R6520) was applied at concentrations of 5 and 10 μmol L^–1^, respectively. The β-receptor agonist isoprenaline (Iso, Sigma-Aldrich, #I5627) was applied at 2 μmol L^–1^. Cells or cardiac slices were incubated with respective mediators for 1 h. All solutions applied in cell culture were sterile filtrated before use.

### Cell Culture

The murine atrial cardiac myocyte cell line HL-1 was kindly provided by William C. Claycomb (Department of Biochemistry and Molecular Biology, LSU Health Sciences Centre, New Orleans, LA, United States) and maintained in Claycomb medium (#51800C) supplemented with 10% fetal bovine serum (#F2442), 100 μmol L^–1^ norepinephrine, 100 μg mL^–1^ penicillin/streptomycin, and 2 mmol L^–1^ L-glutamine at 37°C, 5% CO_2_ and 100% humidity (Claycomb et al., 1998). For experiments, cells were seeded at 50,000–100,000 cells per cm^2^ on cell culture plates coated with 0.02% gelatin and 25 μg mL^–1^ fibronectin. All cell culture reagents were purchased from Sigma–Aldrich, Munich, Germany. After seeding for experiments, cells were incubated in Claycomb medium without norepinephrine to avoid basal adrenergic stimulation and additionally supplemented with 1.8 mmol L^–1^ Ca^2+^ to provide sufficient cadherin binding.

### Mouse Models and Cardiac Slices

The loxP/Cre system with Cre under the alpha myosin-heavy chain promotor was utilized to generate heart-specific Pg deficiency in mice (Schinner et al., 2017), as described before. Mice develop an ACM-like phenotype with progressive cardiac hypertrophy, ventricular dilatation, and fibrosis of the heart muscle (Schinner et al., 2017). Azan stainings were performed as described previously (Schinner et al., 2017) to analyze the pathology of the mouse. The genotype of all animals was evaluated by PCR. Murine cardiac slices were prepared as previously described (Schinner et al., 2017). Alternating slices were used for control and treatments. Age and sex-matched mice were used.

### Antibodies

For immunostaining, the following primary antibodies were applied: polyclonal rabbit anti-DSG2 (#610121, Progen) and monoclonal mouse anti-N-cad (#610921, BD Transduction). As secondary antibodies, Cy2- and Cy3-conjugated goat anti-rabbit or goat anti-mouse secondary antibodies (Dianova, Hamburg, Germany), STAR red goat anti-rabbit IgG (Abberior GmbH, Göttingen, Germany, #2-0012-011-9) or Alexa Fluor 594 goat anti-Mouse IgG (Abcam, Berlin, Germany, #ab150116) for STED were applied. For western blots, monoclonal mouse anti-Dsg1/2 (#61002, Progen, monoclonal mouse anti-N-cad (#610921, BD Transduction), mouse monoclonal anti-PG (#61005. Progen) and monoclonal mouse anti-Tubulin (#ab7291) primary antibodies were used. Polyclonal goat anti-rabbit (#111-035-045, Jackson ImmunoResearch Europe Ltd.) and mouse (#115-035-068, Jackson ImmunoResearch Europe Ltd.) HRP conjugated antibodies were used as secondary bodies.

### Generation of Monoclonal Antibodies Against Phosphoserine 665 of Plakoglobin

A peptide comprising amino acids 656DYRKRVpSVELTNS671 from human PG was synthesized and coupled to OVA (Peps4LS, Heidelberg, Germany). C57BL/6 were immunized subcutaneously and intraperitoneally with a mixture of 50 μg peptide-OVA, 5 nmol CpG oligonucleotide (Tib Molbiol, Berlin), 100 μL PBS, and 100 μL incomplete Freund’s adjuvant. A boost without adjuvant was given 6 weeks after the primary injection. Fusion was performed using standard procedures. Supernatants were tested in a differential ELISA with the phosphorylated peptide or the non-phosphorylated peptide; both coupled to BSA. Monoclonal antibodies that reacted specifically with the phospho-peptide were further analyzed in Western blot. Tissue culture supernatant of PGP (1B8, mouse IgG1, kappa) was used in this study.

### siRNA Knockdown

Murine Dsg2-, N-cad-, or non-targeting siRNA (siDsg2, siN-cad, siNT) (ON-TARGET plus SMARTpool, Thermo Fisher Scientific/Dharmacon, Lafayette, LA, United States) were transfected into HL-1 cells by electroporation (4D-Nucleofector, Lonza, Cologne, Germany) according to manufacturer’s instructions. Confluent HL-1 cells were detached by trypsin and electroporated in Amaxa^TM^ SF solution (Lonza) with 150 μg mL^–1^ siRNA at a concentration of 4 × 10^7^cells mL^–1^ using pulse EN150. Transfection with siNT was performed in parallel. Experiments were performed 72 h after transfection. For each experiment, knockdown efficiency was confirmed by Western blot analysis.

### Western Blot Analysis

Western blot analysis was performed using standard procedures. Confluent HL-1 monolayers were incubated as indicated, washed with PBS, and lyzed in ice-cold SDS-lysis buffer (25 m mol L^–1^ HEPES, 2 m mol L^–1^ EDTA, 25 m mol L^–1^ NaF, 1% SDS, pH7.4) supplemented with a protease inhibitor cocktail (Complete-O, Hoffmann-La Roche). Lysate protein concentration was determined by BCA protein assay kit (Pierce, Thermo Fisher Scientific). Protein samples were denatured in Laemmli buffer at 95°C for 5 min before electrophoresis. After electrophoresis, proteins were transferred on to nitrocellulose membrane (Novex, Thermo Fisher Scientific) using the wet-blot method and blocked in 5% low-fat milk in TBS-T (20 m mol L^–1^ Tris-base, 137 m mol L^–1^ NaCl, 0.0475% Tween, pH 7.6) buffer. Incubation of primary antibodies was performed in 2% bovine serum albumin or 5% low-fat milk in TBS-T buffer at 4°C overnight. Species-matched peroxidase-conjugated secondary antibodies were incubated at room temperature for 120 min in TBS-T and developed with a Fluorchem E (Biozym, Hessisch Oldendorf) using the ECL method.

### Immunostaining

Immunostaining of cultured cardiomyocytes and heart tissue was performed as previously described (Schinner et al., 2017). After incubation with corresponding reagents, cells were washed with PBS, fixed in 2% paraformaldehyde (PFA) in PBS for 10 min, and permeabilized with 0.1% Triton X-100 in PBS for 5 min, following blocking of unspecific binding sites with 3% bovine serum albumin/10% normal goat serum in PBS for 30 min. Primary antibodies diluted in PBS were incubated at 4°C in a humid chamber overnight. Species-matched secondary antibodies were applied in PBS at room temperature in a humid chamber for 1 h. Cardiac tissue was snap frozen in liquid nitrogen and cut with a cryostat (Cryostar NX70, Thermo Fisher Scientific) in 7 μm thin sections. Probes were then heated to 37°C for 8 min, washed in PBS, and fixed in 2% PFA for 10 min. Then slices were permeabilized with 1% Triton-X-100 for 60 min, washed in PBS, and blocked with 3% bovine serum albumin/10% normal goat serum in PBS for 60 min. Species-matched secondary antibodies were applied in PBS at room temperature in a humid chamber for 1 h. Glass coverslips were mounted using 1.5% of the anti-fading reagent n-propyl gallate (Sigma–Aldrich, #P3640000) and 60% glycerol in PBS for Confocal microscopy or ProLong Diamond Antifade Mountant (Invitrogen, Carlsbad, CA, United States, #P36965) for STED microscopy. Staining was evaluated using a Leica SP5 Confocal microscope (Leica, Wetzlar, Germany) equipped with a 63x oil objective using LAS-AF software for image acquisition. STED images were obtained using an expert line setup from Abberior (Abberior Instruments, Göttingen, Germany) equipped with a 100x oil objective using Imspector image acquisition software (Abberior Instruments). ImageJ software (NIH, Bethesda, MD, United States) was applied for image analysis.

### Dissociation Assay

#### In HL-1 Cells

Dissociation assay in HL-1 cardiac myocytes was performed as previously described^7^. In brief, confluent cell monolayers were incubated with dissociation buffer (liberase DH 0.065 U mL^–1^, Hoffmann-La Roche; dispase II 2.5 U mL^–1^, Sigma–Aldrich in HBSS) at 37°C to detach the monolayers from the well bottom. Mechanical stress was applied by rotation of the monolayer on an orbital rotator at 1250 r/min for 5 min. After fixation by 1% PFA, the total number of monolayer fragments was determined using a binocular stereomicroscope (Leica). The resulting number of fragments indirectly correlates with the intercellular adhesion.

#### In Cardiac Slice Cultures

Cardiac myocyte cohesion in murine ventricular tissue was determined as established previously (Schinner et al., 2017). 200 μm thin murine cardiac slices were prepared as described above and transferred to cardiac slice medium (DMEM 1:1 F12 nutrient mixture, 10% serum, 1% minimum essential medium non-essential amino acids and 0.1% 2-mercaptoethanol, 2 mM L-glutamine, 10 U/L penicillin, and 10 μg mL^–1^ streptomycin) (all purchased from Thermo Fisher Scientific) and incubated with indicated mediators at 37°C, 5% CO2. To control variations because of different slice size or location in the ventricle, we used consecutive slices for control and treatment, respectively, and the result of a slice was compared to the respective control slice. After incubation in dissociation buffer, slices were subjected to mechanical stress by repeated pipetting with an electronic pipette. After staining intact viable cardiac myocytes with thiazolyl blue tetrazolium bromide (Sigma–Aldrich), the number of dissociated cardiac myocytes was counted with an inverted microscope (Axio, Carl Zeiss, Oberkochen, Germany) and taken as an indirect measurement for intercellular adhesion.

### Transmission Electron Microscopy

In transmission electron microscopy (TEM), slices were fixed with 1% glutaraldehyde in PBS for 2 h at 4°C. After washing with PBS for three times tissue was post-fixed in 2% osmium tetroxide, dehydrated through an ascending ethanol series subsequently embedded in EPON 812 (Serva Electrophoresis GmBH, Heidelberg, Germany) and cured at 80°C for 24 h. After trimming, ultrathin sections (60 nm) were cut with a diamond knife (DiATOME Electron Microscopy Sciences, Hatfield, PA, United States) and placed on copper/rhodium grids (150 mesh, Plano GmbH, Wetzlar, Germany). Contrasting was performed with a saturated uranyl acetate solution and lead(II)citrate according to standard protocols (Watson, 1958; Reynolds, 1963). Images were acquired using a Libra 120 transmission electron microscope (Carl Zeiss NTS GmbH, Oberkochen, Germany) equipped with a SSCCD camera system (TRS, Olympus, Tokyo, Japan).

For quantitative analysis, 25 ICDs per mouse per condition were analyzed by a blindfolded observer using ImageJ software (NIH). Plaque thickness was determined by measuring from the lateral end of one plaque to the lateral end of the plaque of the adjacent cell and subtracting the measured intercellular space. Measurement was done three times per ICD and averaged to level out differences within one ICD. Area composita length was measured by following the convolution of each ICD.

### Statistics

Results are expressed as mean values ± standard error of mean (SEM). Statistical comparisons were performed using Prism 8 (GraphPad Software). Significance was considered when *P* < 0.05.

## Results

**FIGURE 1:**
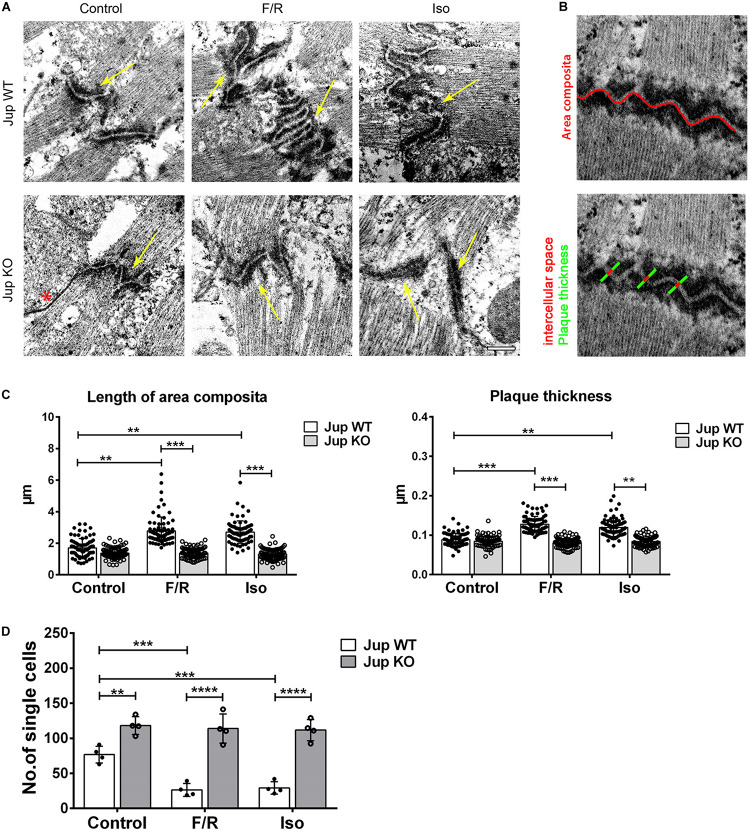
Adrenergic signaling caused ultrastructural changes in ICDs of murine cardiomyocytes and leads to positive adhesiotropy. **(A)** Transmission electron microscopy was performed from cardiac slices derived from the hearts of 12-week-old Jup WT and KO mice (for the sake of terminology here, we used PG gene name “*Jup*” for labeling) treated with F/R and Iso for 1 h and representative images were displayed. Scale bar: 375 nm. * indicates gap junction and the yellow arrow indicates the area composita. *n* = 3 mice per condition. **(B)** Exemplar images of how the analysis of junctional plaque thickness and length of area composita were obtained (as explained in section “Materials and Methods”). **(C)** Bar graphs of plaque thickness and length of area composita measured corresponding to **A**. Every dot corresponds to one ICD, mean ± SEM. **(D)** Dissociation assays performed in cardiac slices derived from Jup WT and KO mice treated with F/R and Iso for 1 h showing an increase of cell adhesion upon adrenergic signaling treatment in Jup WT mice which was completely absent in Jup KO mice. *n* = 4 mice per condition. Two-way repeated measure ANOVA with Tukey’s *post hoc* test was performed. **p < 0.005, ***p < 0.0005, ****p < 0.00005.

Our previous study indicated reorganization of ICDs after adrenergic signaling in cultured cells (Schinner et al., 2017). In this study, we used the cardiomyocyte-specific Pg-depletion model (for the ease of understanding in the complete text we refer these mice as Pg-WT and KO instead of JUP WT and KO written in the figures) to study the role of PG, which was characterized in detail previously (Schinner et al., 2017). These mice develop an ACM-like phenotype with progressive cardiac hypertrophy, ventricular dilatation, and fibrosis of the heart muscle between 6 and 12 weeks (data not shown). In these experiments, we used ventricular cardiac slices cut in sequential order to compare treatments to the control conditions. Cardiac slices from Pg-WT and KO mice treated with F/R and Iso were analyzed by TEM (Figures 1A–C). We observed a slight decrease in the length of area composita in Pg-KO mice compared to the WT mice (1.37 ± 0.13 *vs* 1.7 ± 0.30 μm) which was not statistically significant. When WT mice cardiac slices were treated with F/R, we found an increase in area composita lengths compared to their respective controls (2.78 ± 0.48 after F/R and 2.69 ± 0.18 after Iso *vs* 1.7 ± 0.30 μm). However, in Pg-KO cardiac slices treated with F/R, this increase in area composita length was not found when compared to the respective controls (1.38 ± 0.06 after F/R and 1.31 ± 0.08 after Iso *vs* 1.37 ± 0.13 μm) (Figure 1C). Since we observed some changes in plaque thickness of the area composita, we analyzed plaque thickness (Figures 1B,C). We did not find any change in plaque thickness between WT and Pg-KO mice under control conditions (0.088 ± 0.003 *vs* 0.085 ± 0.006 μm). Nevertheless, we found an increase in plaque thickness in WT cardiac slices treated with F/R and Iso compared to WT controls (0.128 ± 0.012 after F/R and 0.120 ± 0.015 after Iso *vs* 0.088 ± 0.003 μm in control). No changes were observed between Pg-KO cardiac slices treated with and without F/R and Iso (0.080 ± 0.005 after F/R and 0.084 ± 0.003 after Iso *vs* 0.085 ± 0.006 μm in control). The ultrastructural changes after the elevation of cAMP in WT but not in Pg-KO hearts were paralleled by alterations in cardiomyocyte cohesion as revealed by dissociation assays (Figure 1D). In Pg-KO slices compared to WT mice, dissociation assays showed a decrease in cell cohesion, which is evident by the increase in the number of single cells under control conditions. Cardiac slices from WT but not Pg-KO mice treated with F/R and Iso displayed an increase in cell cohesion compared to respective controls.

**FIGURE 2:**
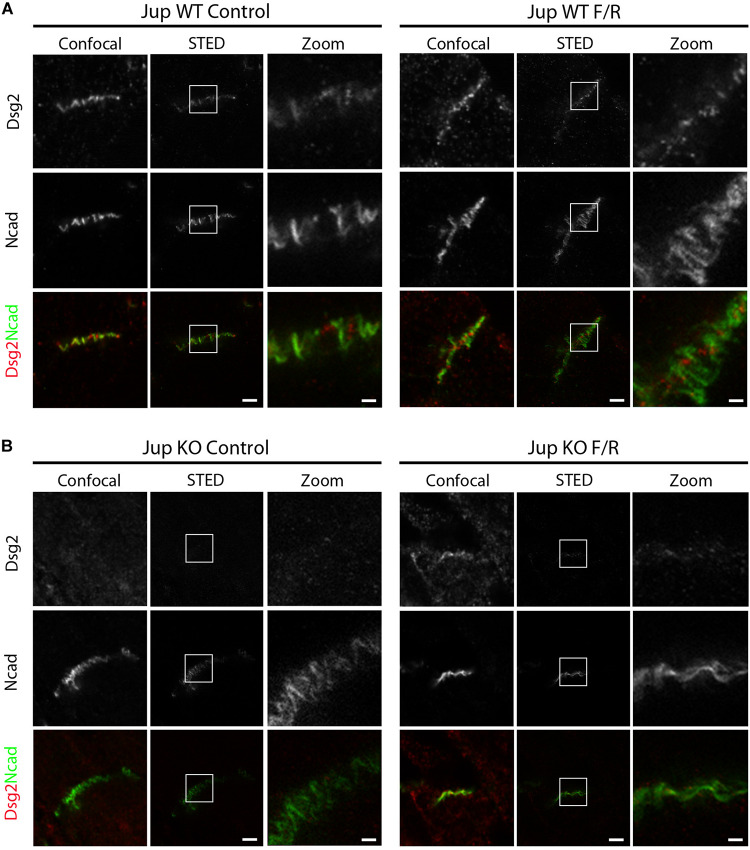
cAMP enhancement in murine cardiac slices leads to changes in the distribution of Dsg2 and N-cad. STED microscopy analysis was performed on cardiac slices derived from hearts of 12-week-old Jup WT and KO mice treated with F/R for 1 h. STED images of ICDs of **(A)** Jup WT and **(B)** Jup KO mice treated with and without F/R for 1 h and stained for Dsg2 (red) and N-cad (green) (*n* = 4 mice per condition). Scale bar: 2 μm. Zoomed image of Dsg2 and N-cad representing the inset was shown in a separate panel.

To further characterize ultrastructural rearrangement within ICDs, we performed STED microscopy in comparison to confocal imaging on murine cardiac slices to visualize distribution of desmosomal protein Dsg2, and AJ protein N-cad. We treated Pg-WT and Pg-KO murine cardiac slices for 1 h in the presence or absence of F/R. Confocal microscopy revealed a wavy pattern of Dsg2 and N-cad distribution in WT ICDs with broad overlap of the two proteins (Figure 2A). However, STED imaging demonstrated that Dsg2 and N-cad are located at distinct parts of the ICD with almost no overlap but intermingled with each other. In contrast, in slices from Pg-KO mice, Dsg2 was strongly reduced as reported previously in this mouse model (Schinner et al., 2017) but N-cad distribution was similar to that of Pg-WT (Figure 2B). In Pg-WT mice cardiac slices, treatment with F/R enhanced staining for Dsg2 and compactly reorganized with N-cad compared to that in control slices. In Pg-deficient slices, there was no effect of F/R compared to controls. The above results clearly established that the elevation of cAMP leads to positive adhesiotropy (Schinner et al., 2017) and that this phenomenon is paralled by ultrastructural reorganization of the ICD.

**FIGURE 3:**
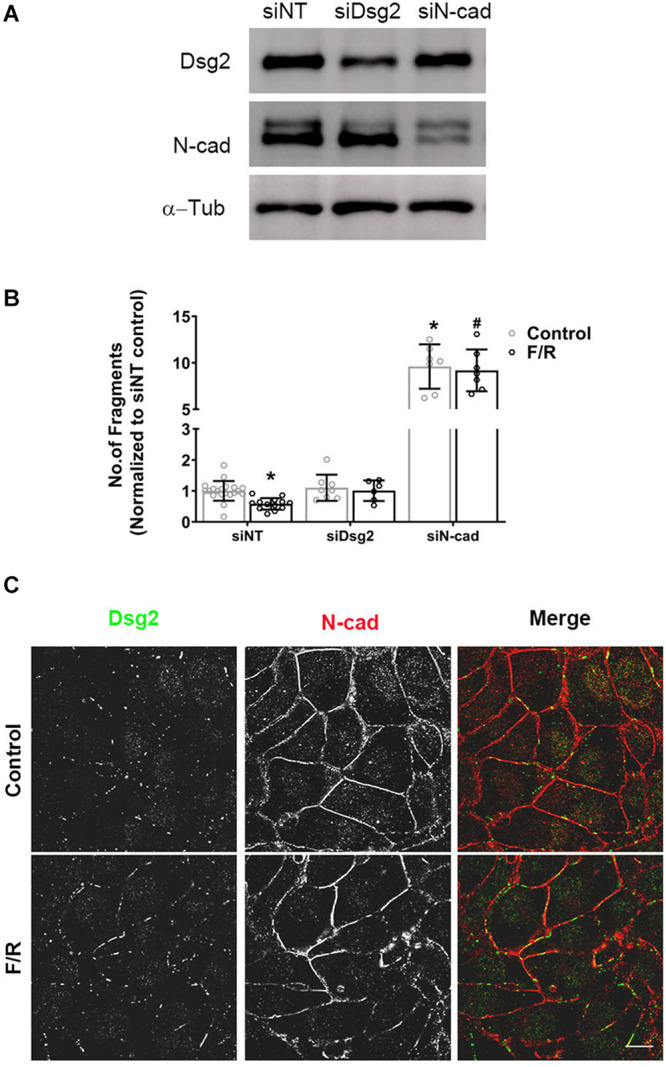
Altered cardiac myocyte cohesion after depletion of Dsg2 and N-cad. Confluent HL-1 cardiac myocyte monolayers were treated with and without F/R for 1 h to increase cAMP. **(A)** Depletion of Dsg2 and N-cad was done by using a siRNA approach, and Western blots were performed to confirm the knockdown of respective proteins. α-Tubulin (α-Tub) was used as a loading control, and representative blots are shown. **(B)** Dissociation assays were performed to determine cell–cell adhesion after depletion of either Dsg2 or N-cad after treating the cells with and without F/R for 1 h, *N* = 3–6 experiments performed in triplicates. Multiple *t*-test with Holm–Sidak multiple comparisons was performed. * is *p* < 0.0001 compared to si NT, #*p* < 0.0001 compared to si NT+F/R. **(C)** Distribution of the desmosomal proteins DSG2 (green) and N-cad (red) was analyzed by immunostaining. *n* = 3, scale bar: 10 μm.

Based on the above results, we further analyzed the role of Dsg2 and N-cad proteins in positive adhesiotropy at molecular level using HL-1 cardiomyocyte cell line. Dsg2 and N-cad were depleted in HL-1 cells using siRNA approach (Figure 3A), and cell–cell cohesion was determined by dispase-based dissociation assays. As observed previously, adrenergic stimulation via F/R treatment strengthened cardiac cell cohesion in siNT (non-target siRNA transfected cells) control cells as indicated by reduced monolayer fragmentation. Depletion of N-cad but not of Dsg2 led to a strong decrease of cell cohesion whereas depletion of either Dsg2 or N-cad abrogated F/R-mediated increase in cell cohesion (Figure 3B), showing the importance of N-cad for positive adhesiotropy. Immunostaining results revealed no changes in N-cad localization to cell borders, whereas an increase in Dsg2 translocation to cell borders was observed after F/R treatment (Figure 3C).

**FIGURE 4:**
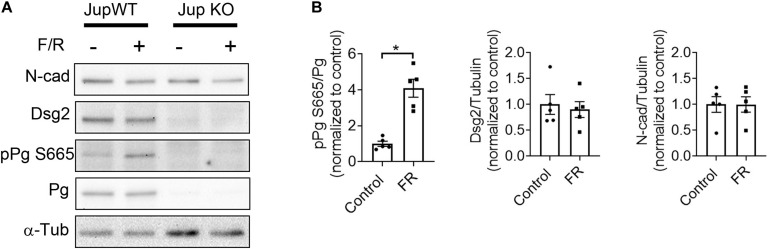
F/R-mediated phosphorylation of PG at serine 665. **(A)** Western blot analysis of ventricular cardiac slices from Jup WT and KO mice treated with F/R for 1 h to analyze phosphorylation state of PG at serine 665. **(B)** Bar graphs depict the mean band intensity by densitometric quantification in WT mice cardiac slices compared to the total protein or α-tubulin (α-tub) and normalized as fold of control ± SEM, *n* = 5 mice per condition. Unpaired student’s *t*-test was performed. * is *p* < 0.05.

Previously, we found positive adhesiotropy to be dependent on the phosphorylation of PG at serine 665, at least in cultured HL-1 cardiomyocytes (Schinner et al., 2017). In this study, to test whether PG phosphorylation also occurs in intact cardiac tissue, we developed an antibody against phosphorylated serine 665 of PG. Western blots performed in cardiac slices from Pg-WT and KO mice, which were treated with F/R, showed an increase in PG serine665 phosphorylation without any changes in the expression of Dsg2 and N-cad in WT hearts (Figure 4). These data combined with our previous observations demonstrate that elevation of cAMP leads to phosphorylation of PG at serine 665 in cardiomyocytes.

## Discussion

Previously, adrenergic signaling-mediated increase in cardiac myocyte cohesion was referred to as positive adhesiotropy (Schinner et al., 2017), and this was shown to occur via PKA-mediated Pg phosphorylation at Serine 665. In the present study, we provide further evidence that this phenomenon is associated with profound ultrastructural changes in the ICDs of murine hearts which require expression of Pg.

Transmission electron microscopy analysis has contributed immensely to our understanding of ICD morphology in healthy and diseased heart (Basso et al., 2006; Bennett et al., 2006; Manring et al., 2018). Therefore, in this study, we analyzed the cardiac tissues from WT and Pg-KO mice utilizing TEM. By now it is well-established that mixing of AJ and desmosomal components at the ICD is typical, which is reffered to as area composita (Franke et al., 2006, 2007; Pieperhoff and Franke, 2007). Therefore, we used actomyosin filaments to determine ICD segments as area composita, of which we measured the length and plaque thickness. We observed a substantial increase in the area composita length in WT cardiac myocytes after F/R and Iso treatment compared to controls. Similarly, we also observed thickening of the plaque region in WT cardiac myocyte ICDs after augmenting cAMP. Under basal conditions, in Pg-KO hearts, we did not observe any changes in area composita length or plaque thickness compared to WT hearts. However, when F/R or Iso were used to increase cAMP, Pg-KO hearts did not respond with changes in area composita length or plaque thickness. This may be explained by the requirement of PG phosphorylation for the positive adhesiotropic response (Schinner et al., 2017). In ACM patients, Basso et al. (2006) found a substantial remodeling of ICD in ACM patients, where they found abnormally located desmosomes and pale internal plaques. Absence of changes in ICDs in the Pg-KO mice can be attributed to the upregulation of β-catenin in these mice as was reported earlier (Li et al., 2011; Schinner et al., 2017). Since β-catenin is also known to interact with N-cad, this may be sufficient to maintain N-cad at ICDs. In line with this, double knockout of β-catenin and PG in the heart led to the loss of N-cad from the ICD (Swope et al., 2012).

A recent study utilizing proximity proteomics in primary cardiomyocytes showed that Dsg2 and PG are found in close proximity to N-cad (Li et al., 2019). Similarly, STED imaging in our study demonstrated that in WT murine hearts, N-cad and Dsg2 are localized in distinct sections of the ICD but intermingled with each other. Probably they are connected via PG. Further experiments would be needed to fully understand these observations. In fact, it is very well known that in ICDs both desmosomes and AJs are intermingled and this region is termed as area composita. In the Pg-KO mice, Dsg2 was confined to small dots at the ICD as revealed by STED imaging whereas N-cad distribution was similar to WT. Moreover, in Pg-KO hearts, increased cAMP was not effective to localize Dsg2 to ICDs, suggesting that PG is more important for Dsg2 function. Before, it has been reported that in Pg-KO cardiac myocytes, Dsg2 is expressed at low levels whereas N-cad has sufficient expression (Li et al., 2011). Our results are in line with the already published data but STED microscopy analysis of ICDs for Dsg2 and N-cad to our knowledge was not analyzed until now.

In recent times, several studies supported that most ICD components structurally interact with each other (Vermij et al., 2017; Zhao et al., 2019). In our previous study (Schinner et al., 2017), we focused on the role of Dsg2 in positive adhesiotropy as we found that N-cad localization in HL-1 cells did not change in response to enhanced cAMP levels. On the other hand, there were also studies that showed enhancement of N-cad staining at cell borders upon cAMP treatment during cardiac myocyte junction assembly (Somekawa et al., 2005) and PG, the major protein involved in positive adhesiotropy, is also known to bind to N-cad. In addition, studies performed in the past also established that AJ formation organized by N-cad is essential for GJ assembly in cardiomyocytes (Hertig et al., 1996a,b; Kostin et al., 1999; Kostetskii et al., 2005). Based on these observations, we investigated if the observed ultrastrutural changes by adrenergic signaling in ICD, due to changes in the reorganization of Dsg2 and N-cad, leads to any changes in the way Dsg2 and/or N-cad function and there by affecting positive adhesiotropy in cardiomyocaytes. For this purpose, we utilized HL-1 cells.

siRNA-mediated N-cad knockdown in HL-1 cells resulted in a severe decrease in cell cohesion and ablated the F/R effect. In contrast, knockdown of Dsg2 did not change basal cell adhesion but abrogated the F/R effect as we observed previously (Schinner et al., 2017). This shows that N-cad might be necessary for both basal and cAMP-mediated strengthening of cell cohesion whereas Dsg2 seems to be essential for positive adhesiotropy. Immunostaining did not detect any changes in N-cad localization after F/R treatment whereas Dsg2 was found to be enriched at cell–cell contacts. These results support the idea that N-cad and Dsg2 are important for intercellular adhesion. This interdependency of proteins in the ICD and changes in their interaction under pathological conditions has been proposed to also cause ultrastructural changes of ICD (Zemljic-Harpf et al., 2007; Ye et al., 2014). Utilizing HL-1 cells for mechanistic approaches is not always desirable as their cell–cell contacts might not be comparable to more mature cardiomyocytes of the neonates or adult mice as observed in our previous study (Schinner et al., 2017). Nevertheless, we previously utilized HL-1 cells and showed that adrenergic signaling enhances cardiomyocyte adhesion via PKA mediated PG phosphorylation at serine 665. In this study, for the first time in cardiac slices of mice, we show that elevation of cAMP leads to PG phosphorylation at serine 665 which is paralleled by enhanced ultrastructural changes in the ICD. Together with our previous findings showing that PG serine 665 phosphorylation is required for cAMP-mediated stabilization of Dsg2 adhesion and Dsg2 recruitment to cell junctions, the new data strongly suggest that PG phosphorylation strengthens cardiomycyte adhesion via ultrastrutural alterations at ICD.

## Conclusion

We report here that in positive adhesiotropy, strengthening of cardiomyocyte cohesion in intact hearts is accompanied by profound alterations of the ICD ultrastructure, all of which are dependent on PG expression. Our data from the Pg KO mice can be correlated to the ACM patients with mutations in Pg gene, where probably similar mechanisms might exist. We strongly believe that further studies utilizing cardiomyocytes derived from ACM patients iPSCs can enhance our understanding on how the ICDs are aberrated in patients with different desmosomal protein mutations and whether adrenergic signaling can positively modulate these aberrations in these patients or not and thus may also be relevant for therapeutically targeting ACM.

## Data Availability Statement

All datasets generated for this study are included in the article/Supplementary Material.

## Ethics Statement

Handling, breeding, and sacrificing of mice for scientific purposes were performed under approval of regulations of the Regierung von Oberbayern (Gz.: 55.2-1-54-2532-139-2014).

## Author Contributions

SY, EK, DE, HF, and DK participated, acquired, and analyzed the data. SY and JW drafted the manuscript and made critical revision of the manuscript for important intellectual content. JW supervised and designed the research.

## Conflict of Interest

The authors declare that the research was conducted in the absence of any commercial or financial relationships that could be construed as a potential conflict of interest.

## Supplementary Material

FIGURE S1 H& E and Azan staining of JupWT and KO mice hearts. Representative images of H&E and Azan stainings performed in 12-week-old JupWT and KO mice hearts to reveal the morphology and fibrosis detectable in Jup KO but not WT mice. Zoomed areas of boxes were shown in below panel. Arrows indicate fibrotic areas. ^∗^ indicates artery.
