# Supplementary figures and images for: Adrenergic Signaling-Induced Ultrastructural Strengthening of Intercalated Discs via Plakoglobin Is Crucial for Positive Adhesiotropy in Murine Cardiomyocytes
Source: Front Physiol. 2020 May 21;11:430. doi: 10.3389/fphys.2020.00430 (PMC7253624; doi:10.3389/fphys.2020.00430)

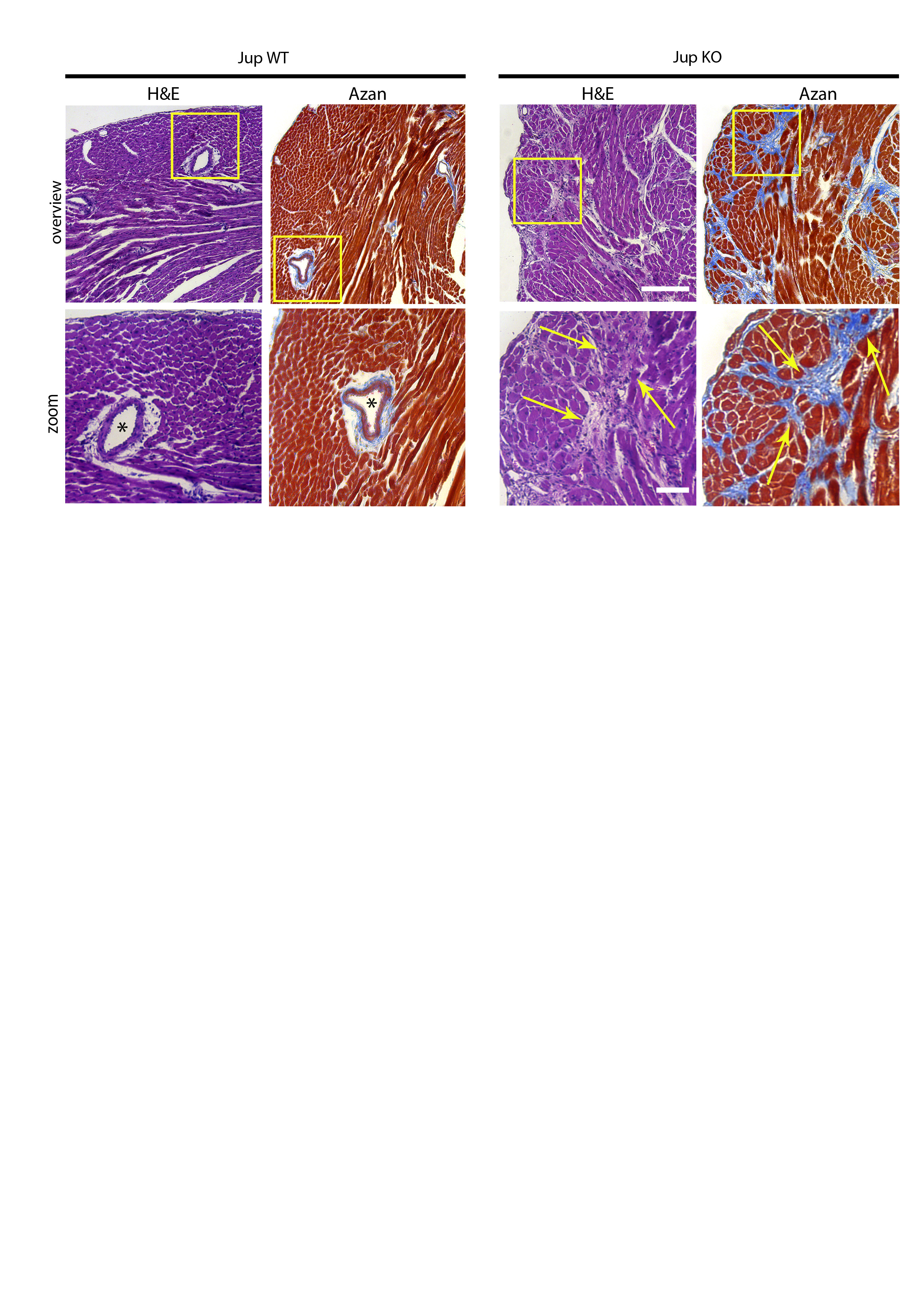

Supplement: FIGURE S1 — H& E and Azan staining of JupWT and KO mice hearts. Representative images of H&E and Azan stainings performed in 12-week-old JupWT and KO mice hearts to reveal the morphology and fibrosis detectable in Jup KO but not WT mice. Zoomed areas of boxes were shown in below panel. Arrows indicate fibrotic areas. ∗ indicates artery. [file Image_1.JPEG]
